# Viral and Bacterial Co-Infections in the Lungs: Dangerous Liaisons

**DOI:** 10.3390/v13091725

**Published:** 2021-08-30

**Authors:** Justine Oliva, Olivier Terrier

**Affiliations:** CIRI, Centre International de Recherche en Infectiologie, (Team VirPath), University Lyon, Inserm, U1111, Université Claude Bernard Lyon 1, CNRS, UMR5308, ENS de Lyon, F-69007 Lyon, France; justine.oliva@univ-lyon1.fr

**Keywords:** co-infections, superinfections, respiratory infections, respiratory viruses, influenza virus, respiratory syncytial virus, SARS-CoV-2

## Abstract

Respiratory tract infections constitute a significant public health problem, with a therapeutic arsenal that remains relatively limited and that is threatened by the emergence of antiviral and/or antibiotic resistance. Viral–bacterial co-infections are very often associated with the severity of these respiratory infections and have been explored mainly in the context of bacterial superinfections following primary influenza infection. This review summarizes our current knowledge of the mechanisms underlying these co-infections between respiratory viruses (influenza viruses, RSV, and SARS-CoV-2) and bacteria, at both the physiological and immunological levels. This review also explores the importance of the microbiome and the pathological context in the evolution of these respiratory tract co-infections and presents the different in vitro and in vivo experimental models available. A better understanding of the complex functional interactions between viruses/bacteria and host cells will allow the development of new, specific, and more effective diagnostic and therapeutic approaches.

## 1. Respiratory Tract Infections, Their Etiological Agents, and the Weight of Viral–Bacterial Co-Infections

Respiratory tract infections (RTI) constitute a leading cause of morbidity and mortality worldwide, in children and adults, accounting for approximately 3 to 5 million deaths per year, with a considerable impact on public health and society, and at the economic level [1,2,3]. RTIs refer to a range of infections confined to the upper respiratory tract (rhinitis, sinusitis, pharyngitis, or tracheitis) and/or lower respiratory tract (mainly bronchitis and pneumonia), implicating microorganisms, including viruses, bacteria, and fungi. The nature of the pathogens involved is very heterogeneous and broad, reflecting many very different infection scenarios. [4]. Co-infections can be defined as concomitant infections, while superinfections are sequential infections by two different pathogens. The most documented scenario in the literature is that of bacterial superinfections following primary viral infection. [5]. The burden of bacterial co-infections and superinfections can be quite different depending on the nature of the primary viral infection.

### 1.1. Influenza Viruses

Post-influenza bacterial pneumonia is known to play a significant role in the morbidity and mortality associated with both seasonal and pandemic influenza virus illness [6]. An important part of pandemic prevention and management is understanding the relationship between influenza infection and secondary bacterial infection [7,8,9,10]. Much of our knowledge about the severity of post-influenza bacterial pneumonia comes from retrospective studies in the context of past influenza pandemics. Most deaths in the 1918–1919 influenza pandemic likely resulted directly from secondary pneumonia caused by common upper respiratory tract bacteria. Lung tissue samples suggest that most of the estimated 20–60 million deaths were from bacterial superinfections rather than from direct effects of the virus. However, less substantial data from the subsequent influenza pandemics are consistent with these findings [11]. During seasonal epidemics, influenza bacterial co-infection is associated with increases in hospital admission. One in four patients admitted to ICU with severe influenza A infection presented bacterial or viral co-infection [12]. In a national survey in the US during the 2003–2004 influenza season, infectious disease specialists observed a 1.6% and 2% rate of bacterial complications in adult and pediatric patients, respectively [13]. Systemic review and meta-analysis revealed that the frequency of bacterial co-infection was highly variable, ranging from 2% to 65%. In accordance with other studies [14,15], the most prevalent coinfecting bacterial species were *Streptococcus pneumoniae* and *Staphylococcus aureus*, which accounted for 35% (95% CI, 14–56%) and 28% (95% CI, 16–40%) of infections, respectively. A wide range of other pathogens, such as *Haemophilus pneumoniae* or *Klebsiella pneumoniae*, caused the remaining infections [16]. Teng and collaborators performed a retrospective, observational study during the eight influenza seasons from 2010 to 2018. Of the 209 influenza-associated pneumonia-admitted patients, 41 (19.6%) were identified with community-acquired bacterial co-infections, mainly with *S. aureus*. This phenomenon was frequently observed in influenza-associated pneumonia, but no risk factor has been identified so far. Bacterial co-infection is likely to predict severity and is an independent risk factor for in-hospital mortality. Furthermore, mixed infection with *S. aureus* and influenza has frequently led to a lethal synergism [17].

### 1.2. Respiratory Syncytial Virus

RSV is the most common cause of bronchiolitis in children less than 1-year-old. RSV is also responsible for acute lower respiratory tract infection in the elderly and in immunocompromised adults. The disease is often associated with a simultaneous or secondary bacterial infection. Co-infection with RSV and bacteria is well described, but studies to decipher the underlying molecular mechanisms remain limited (reviewed in [18,19]). Various studies of RSV-infected patients in the hospital revealed an association with a lower respiratory tract bacterial co-infection, ranging from 17.5 to 44% of patients positive for both RSV and bacterial co-infection. The most common bacteria isolated were *S. pneumoniae* and *H. influenzae* [20,21,22]. However, recent studies suggested a most prevalent association between RSV and *S. aureus*, especially the methicillin-resistant *S. aureus* (MRSA) [19]. Bacterial co-infection combined with RSV infection often correlates with more severe disease in susceptible people than simple RSV infection [21,23].

### 1.3. SARS-CoV-2

As of the 9 August 2021, 19 months after its discovery, SARS-CoV-2 has rapidly become a major global pathogen, with the COVID-19 pandemic affecting more than 202 million people and causing more than 4,288,134 deaths worldwide [24]. A large multicenter prospective cohort study recently demonstrated that microbiologically confirmed bacterial infections, mainly secondary, were rare (less than 2.3%) in patients admitted to hospital with COVID-19 during the first wave of the pandemic in the UK [25]. This result agrees with several previous observational studies and meta-analyses that have reported a low frequency of bacterial co-infections in people admitted to hospital for COVID-19 [26,27,28,29]. Recently, Langford and collaborators performed a meta-analysis with 3338 COVID-19 patients for the evaluation of bacterial co-infection. This phenomenon occurred in 3.5% of patients (95% CI 0.4–6.7%), but secondary bacterial infection was also observed in 14.3% of patients (95% CI 9.6–18.9%). Generally, the proportion of COVID-19 patients presenting bacterial infection was 6.9% (95% CI 4.3–9.5%). However, bacterial co-infection was more often observed in critically ill patients (8.1%, 95% CI 2.3–13.8%). A low frequency of bacterial co-infection was observed in hospitalized patients and may not require anti-bacterial treatment [29]. Similar results were observed in other meta-analysis studies. *S. pneumoniae*, *S. aureus*, *Pseudomonas aeruginosa*, and *Escherichia coli* were mainly identified, especially in critically ill patients. COVID-19 patients with community-acquired co-infections and hospital-acquired superinfections developed the worst outcomes compared to simple-infected patients [30]. Lansbury and collaborators also observed a low frequency of co-infection in COVID-19 patients, with a higher percentage in ICU patients. The most common bacteria detected were *Mycoplasma pneumonia*, *P. aeruginosa*, and *H. influenzae* [28]. Interestingly, a recent study that aimed to determine the incidence of bacterial co-infections in 925 hospitalized COVID-19 patients found that such bacterial co-infections are rare [26]. This finding agrees with earlier retrospective observational studies that reported a low frequency of bacterial co-infection in early COVID-19 hospitalized individuals [27,31]. By contrast, other studies claim that bacterial (and fungal) co-infections exist in severe COVID-19 patients and include *Acinetobacter baumannii* and *K. pneumoniae* [32]. Nevertheless, all clinical data show that the bacterial or fungal co-infection rate of SARS-CoV-2–infected patients is lower than in influenza-virus-infected ones. This may be due to an underreporting issue, an extensive use of antibiotics, or the implementation of control measures limiting the spread of several respiratory pathogens. So far, the importance of co-infection in COVID-19 patients and its effects on pathogenesis remains poorly described. 

## 2. Mechanisms Involved in Co-Infections/Superinfections

Various mechanisms are involved in respiratory co-infections and superinfections. For a long time, the impact of viral infection on the epithelial barrier was considered the primary cause of bacterial superinfection. Recently, several studies demonstrated that the antiviral immune response also plays a role in mixed infections. Although presented separately in this review (for clarity purposes), physiological and immunological mechanisms are concomitant and closely associated.

### 2.1. Physiological Mechanisms

#### 2.1.1. Epithelium Damage

The epithelium has a crucial role in preventing the invasion of inhaled pathogens and particles. Epithelial cells, assembled in a pseudostratified structure through tight junctions, create an impermeable barrier for pathogens [33]. A sharp decrease of transepithelial resistance and modification of cell morphology were observed in human primary epithelial cells (HAE) cultured at the air–liquid interface after SARS-CoV-2 infection, suggesting a disruption of epithelium integrity [34]. Comparable observations were observed with influenza and RSV using similar in vitro models. A correlation was also noticed in animal models with desquamation, loss of cilia, immune cells infiltration, and necrosis observed after viral infection [35,36,37,38,39]. A dysfunction of tight junctions and cytoskeleton are frequently observed after single viral infection. RSV and influenza viruses can decrease tight junctions by directly or indirectly targeting the proteins involved, such as claudin, occludin, or ZO-1, and induce an F-actin cytoskeleton rearrangement leading to cell morphology modifications [40,41,42]. In vitro and in vivo co-infection studies demonstrated that the epithelium damage caused by respiratory viruses constitutes one of the causes leading to secondary infection. Primary influenza or RSV infection induce epithelium damage that leads to a higher susceptibility to *S. aureus* or *S. pneumoniae* in animal models [43,44,45,46,47,48]. Viral-induced cell apoptosis can also be responsible for the loss of the epithelium barrier through various mechanisms such as the FasL/TRAIL pathway [49,50,51]. Although not being specifically studied, some works suggest a correlation between virus-induced apoptosis/necrosis and higher susceptibility to bacterial superinfection, with cell debris improving bacterial adhesion and invasion [52]. 

#### 2.1.2. Modification of Airway Function

Virally induced modifications of airway function are also responsible for respiratory co-infections. The epithelium can thwart infection thanks to mucociliary clearance, which regroups two essential mechanisms: (i) the production of mucus, a multi-component secretion, which entraps the inhaled pathogens [53] and (ii) the presence of motile cilia recovering the airway with continuous beating [54]. Epithelial cells are well known to produce mucus after infection to reduce the infection by influenza, RSV, or SARS-CoV-2. However, this leads to airway obstruction, reflecting the diminution of pulmonary capacity observed in patients [53,55]. RSV and influenza viruses are also known to increase mucus-associated protein, such as mucin 5 after infection [56,57]. Respiratory viruses mainly target ciliated epithelial cells, inducing cell death and thus, cilia loss. So far, no study has explicitly focused on the molecular mechanisms of mucociliary clearance during co-infection. 

#### 2.1.3. Enhancement of Bacterial Adhesion after Viral Infection

Primary viral infection can also increase bacterial adherence in the respiratory tract. RSV infection increases the adhesion and the virulence of *S. pneumoniae* on epithelial cells through direct binding of G glycoprotein to bacterial components. The G glycoprotein anchors at the cell membrane after infection and acts as a bacterial receptor [58,59,60]. A transcriptomic study revealed that RSV increased adherence molecules on RSV-infected cell surfaces, such as CD47, leading to an increased *S. pneumoniae* adhesion [61]. The influenza virus also enhanced the adhesion of *S. aureus* or *S. pneumoniae* in various models by increasing fibrinogen, modifications of glycoproteins, and sialic acids on the infected cell membrane [62,63]. Platelet-activating factor receptor (PAF-R) has also been described to interact with bacteria, promoting superinfections [64,65,66]. The influenza glycoproteins hemagglutinin and neuraminidase also promote bacterial adhesion, acting as, or exposing bacterial receptors [67,68,69,70,71,72,73]. So far, the effect of SARS-CoV-2 on bacterial adherence during superinfection is poorly described; however, results suggested that another human coronavirus (HCoV-NL63) enhanced *S. pneumoniae* superinfection in LLC-MK2 and HAE cells but not for other bacteria such as *S. aureus*, *H. influenza*, or *P. aeruginosa* [74].

#### 2.1.4. Repair Delay after Viral Infection

Various murine models of superinfection with influenza and *S. pneumoniae* or *S. aureus* demonstrated a lethal synergism when the bacteria were inoculated at seven days post-influenza infection, suggesting that superinfection enhances pathology severity in the later stage of viral infection, during the repair processes. Impairment of repair cell response, especially from macrophages and epithelial cells, was observed, with a decrease in cell regeneration and modification of homeostasis signaling pathway [57,75,76,77]. RSV can interfere with repair mechanisms by increasing the production of MMPs or growth factors, leading to enhanced fibrosis [78]. Transcriptional profiling of a superinfected mouse model with influenza virus and *S. pneumoniae* revealed an increase in epithelial cell proliferation and epithelium-repair 48hpi. Moreover, a correlation was observed between gene up-regulation and disease severity, suggesting that alteration of the repair mechanism was involved in superinfection [79]. Major and collaborators recently demonstrated that types I and III IFN induced by the influenza virus delayed epithelial cell proliferation during the repair stage. Increased apoptosis through activation of p53 and cell cycle alteration was noticed, leading to cell differentiation and growth inhibition and delayed epithelium repair. Influenza-infected Ifnlr1−/− mice had better survival after superinfection with *S. pneumoniae*, suggesting that IFN-λ induced after viral infection delays epithelium repair and contributes to secondary bacterial infection [80]. 

**Figure 1:**
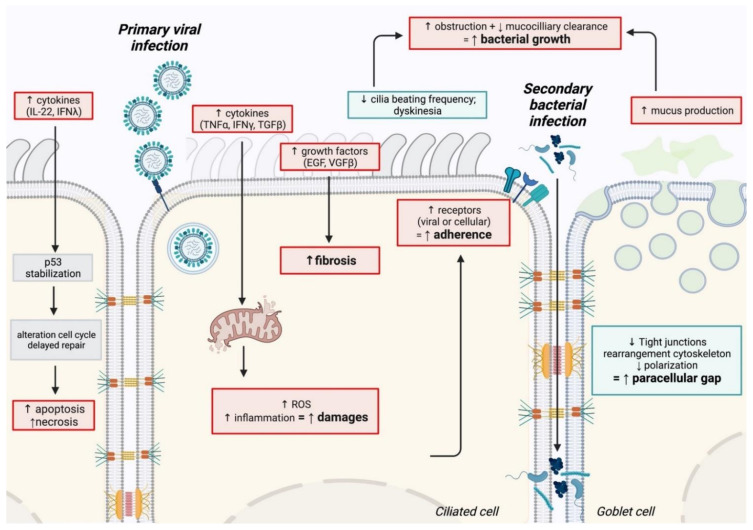
Schematic representation of the physiological mechanisms associated with bacterial superinfections. The impact of the primary viral infection on the integrity and functionality of the epithelium (epithelial damage, repair delay) contributes to creating a favorable environment for the establishment of a secondary bacterial infection. Created with BioRender.com.

The physiological mechanisms underlying co-infections are summarized in Figure 1.

### 2.2. Immunological Mechanisms

Recent studies demonstrated that respiratory superinfection is associated with an alteration of the immune response, especially with a lessening of the numbers and functions of innate and adaptive cells [81,82]. 

#### 2.2.1. Innate Immunity

The first line of defense is innate immune cells such as airway macrophages (AM), monocytes, neutrophils, natural killer (NK) cells, dendritic cells (DC), and epithelial cells [83,84,85,86], sense respiratory pathogens by different pattern recognition receptors (PRRs), such as the Toll-like receptors (TLRs) (TLR3 and TLR7/8 and, to a lesser extent, TLR4, TLR2, and TLR9), retinoic acid-inducible gene I (RIG-I), and NOD-like receptors (NLRs), especially inflammasome NLRP3 [87,88,89,90,91,92,93]. Their engagement induces the activation of transcription factors (IRF3, IRF7, and NF-κB), eliciting the production of pro-inflammatory effectors such as type I and III interferons (IFN), pro-inflammatory cytokines (IL-1β, IL-6, IL-18, and TNFα) and chemokines (IP-10, RANTES, CCL2, MIP-1, and IL-8) [94,95,96,97,98,99,100]. Primary viral infection by influenza led to a downregulation of various TLRs, such as TLR2, TLR4, and TLR5, inducing an irresponsiveness to secondary bacterial infection and thus a decreased bacterial clearance [101]. PRRs, such as TLR9, TLR3, or RIG-I, also had a detrimental role in mixed influenza or RSV/bacterial infection [102,103]. Little is known about the effect of co-infection on inflammasome, but primary viral infection seemed to influence its activation and the production of IL-1β, crucial for bacterial clearance [104].

Superinfection after viral infection impairs the recruitment and/or the functions of innate immune cells. Influenza virus infection in mice led to a depletion of 90% of alveolar macrophages, and the remaining 10% presented a necrotic profile associated with increased susceptibility to *S. pneumoniae* [105,106]. Other studies suggested dysfunctional macrophages rather than impaired recruitment led to bacterial superinfection, with a dampened activation mediated with STAT2-dependent pathway, or decreased phagocytosis and apoptosis caused by the downregulation of the scavenger receptor MARCO [107,108]. Various studies demonstrated the impairment of ROS production after RSV or influenza infection, promoting secondary bacterial infection [109,110,111]. Contrary to alveolar macrophages, neutrophilia are observed after RSV and influenza infection. Surprisingly, neutrophil depletion was not associated with susceptibility to secondary infection, suggesting impaired function was responsible for bacterial clearance decrease [112,113,114]. Primary viral infection dampened neutrophil killing processes, such as ROS production and phagocytosis, leading to decreased bacterial clearance [86,111,115]. Despite an increased formation of neutrophil extracellular traps (NETs) after RSV and influenza infection, their dysregulated functions led to an inability to control secondary bacterial infection by *S. pneumoniae* [116,117]. 

Other innate cells are modulated during co-infections. Inflammatory monocytes promote epithelium injury through an influenza-induced TRAIL-dependent mechanism, promoting secondary infection [118]. Influenza-induced NK cell impairment leads to uncontrolled proliferation of *S. aureus* [119]. Moreover, activation of dendritic cells (DCs) is usually inhibited after influenza and RSV infection. Although the role of DCs in co-infections is still unknown, an alteration of their functions might decrease T cells response and increased susceptibility to co-infection [120,121,122]. Little is known about the effect of SARS-CoV-2 during co-infections or its role on innate immune cells. Various clinical and in vivo studies described an alteration of innate immune cells, characterized by an increased/decreased number of immature phenotypes that could lead to superinfection [123,124].

Innate immune cells are also significant producers of cytokines and chemokines, with a crucial role in controlling and resolving infection. Influenza infection was shown to dampen the production of pro-inflammatory IL-1β, IL-6, and TNF-α, leading to a decreased bacterial clearance [119]. The importance of balance between IL-13 and IFN-γ was brought to light during dual infection—decreased IL-13 and its IFN-γ-inhibitory property increased bacterial susceptibility [125]. A crucial role of IL-10, an anti-inflammatory cytokine secreted in the later stage of influenza or RSV infection, was also observed in superinfections. A regulation of neutrophil and macrophage activity was also observed during superinfection, suggesting that IL-10 inhibited innate immune response against a second pathogen [115,126,127]. Analysis of transcriptomic signatures revealed an increase of IP-10 (CXCL-10) during co-infection with RSV or influenza and bacteria, promoting the recruitment of immune cells and contributing to lung pathology [128,129]. Type I interferons (IFN-α/β) have a crucial role in early antiviral immunity through the induction of hundreds of interferon-stimulated genes (ISGs). Still, distinct effects were also observed on the outcome of bacterial infection [130]. Various studies using transgenic mouse models demonstrated the detrimental role of type I IFN in subsequent bacterial infection following virus infection, with an inhibition of neutrophil recruitment and function and type 17 immune response [131,132,133,134,135]. Shepardson and collaborators noticed a distinct role of type I IFNS, with IFN-β reducing MRSA susceptibility in the pre-clinical stage of influenza infection and IFN-α promoting the susceptibility in the clinical stage [136]. Co-infection with influenza virus and *S. pneumoniae* increased the expression of many miRNAs, such as miRNA-200a-3p, leading to an inhibition of the JAK-STAT inhibitor SOCS6, and this might increase the production of type I-IFNs exacerbating their detrimental effect [129]. Recent studies demonstrated that type III interferons are crucial in promoting superinfections, with similar mechanisms to type I IFN [137,138].

#### 2.2.2. Adaptive immunity

Adaptive response, including T CD8+ and T CD4+ cell and antibody responses, is essential to resolving the respiratory infection. Numerous studies in mouse models with influenza virus and *S. aureus* demonstrated the importance of type 17 response, especially the production of IL-17 and IL-22, for efficient bacterial clearance. A recent study demonstrated that IL-22BP−/− mice are protected during influenza and bacterial super-infection, suggesting that IL-22-binding protein has a pro-inflammatory role and impairs epithelial barrier function likely through interaction with IL-22 [139]. Mixed infections decreased cytokine production by Th17 and γδT cells, and thus impaired bacterial clearance. Type I IFNs impaired the activation of Th17 through a decreased IL-23 and IL-1β production by dendritic cells. STAT1 also had a role in dampening type 17 response, as STAT1−/− mice better controlled secondary bacterial infection than WT mice. Finally, IL-27, known to interfere with type 17 response, was enhanced in mixed infection [132,140,141]. A detrimental role of Th1 cells was observed in CD4-depleted CD8α−/− mice, with an improved bacterial clearance related to decreasing production of IFN-γ [108]. Although no study has demonstrated their role so far, regulatory T cells (Tregs) might be responsible for IL-10 production in the later stage of infection, thus increasing susceptibility to bacterial infection. Tregs could inhibit T CD8+ and T CD4+ cell functions and/or recruitment [126,142]. Blevins and collaborators noticed a depletion of T CD8+ cells and a decrease of IFN-γ and TNF-α by remaining T CD8+ cells, which may lead to secondary infection. T CD8+ cells also have a detrimental role on other cells, as IFN-γ producing T CD8+ cells inhibited the anti-bacterial function of macrophages in the recovery stage [108]. So far, the role of humoral response in co-infection is still poorly understood, and divergent results have been observed. Wu and collaborators noticed a dampened T CD4+ cells, associated with a decreased B-cell- and antibody response, whereas Wolf and collaborators observed an increase in antibody response [143,144]. Recently, the role of innate-like unconventional cells such as γδT cells, mucosal-associated invariant T (MAIT) cells, or invariant natural killer T (iNKT) cells, situated at the interface of innate and adaptive immunity, was described during co-infection. Post-influenza infection by *S. pneumoniae* induced a decrease of IFN- γ-producing iNKT as well as IL-17-producing γδT cells, through a type I IFN-dependent manner, increasing the susceptibility to secondary bacterial infection [133,145,146].

#### 2.2.3. Other mechanisms

The dysregulation of immune response and the alteration of airway epithelium aside, other mechanisms are also involved in respiratory co-infections. For example, McCauley and collaborators demonstrated that the accessory influenza protein PB1-F2, especially the C-terminal domain of the protein, increased the inflammation and the frequency/severity of superinfections. Another study also demonstrated that PB1-F2 contributed to inflammation through a mitochondria-mediated cell death but was restricted to the lab strain PR8 [147,148,149]. 

**Figure 2:**
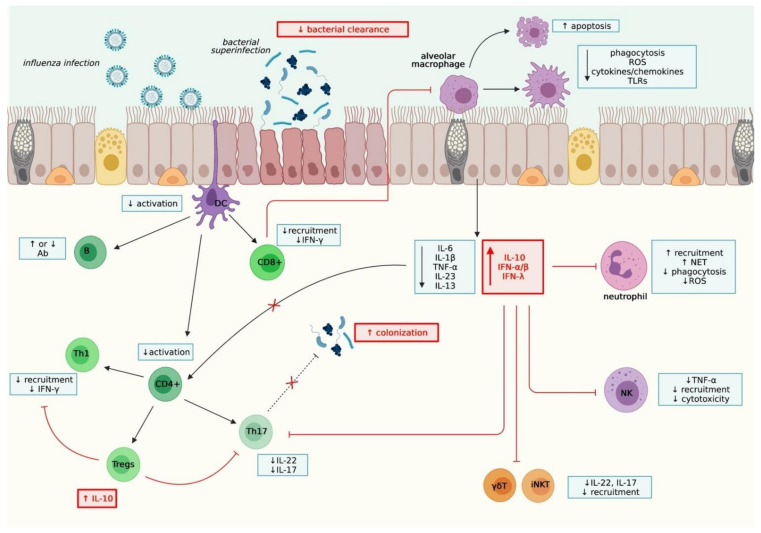
Schematic representation of the immunological mechanisms underlying virus-bacteria co-infections. These mechanisms involve different cell types, in closely related innate and adaptive immune responses. The regulation of the magnitude and timing of these responses plays an essential role in the balance between colonization and bacterial clearance. Created with Biorender.com.

The overall immunological mechanisms underlying co-infections are summarized in Figure 2.

## 3. Viral and Bacterial Co-Infections in the Context of Chronic Respiratory Diseases

There is now a broad consensus that airway infections constitute a significant risk for patients with chronic respiratory diseases, such as asthma, chronic obstructive pulmonary disease (COPD), or cystic fibrosis (CF) [150,151]. Nevertheless, the underlying mechanisms involving co-infections between bacteria and viruses are still relatively poorly characterized. 

In CF, an autosomal recessive disease affecting nearly 70,000 people worldwide, respiratory infections play an essential role in the development and progression of lung disease, *P. aeruginosa* and *S. aureus* being the predominant primary causative agents [152,153,154]. However, the underlying mechanisms that explain the severity of infections in CF patients are still not well understood. A large majority of CF patients die due to respiratory failure caused by chronic bacterial infection and concomitant airway inflammation [150]. Several factors are well known to favor bacterial bronchial colonization, such as mucus hyperviscosity, increased bacterial adhesion via the overproduction of ganglioside-type cell receptors, and a decrease in immune defenses. In this context, early infection of the airways with bacteria such as *S. aureus* or *S. pneumoniae*, followed by later chronic infection with *P. aeruginosa*, is a major clinical problem for patients, as these infections are very quickly accompanied by severe deterioration in lung function [150]. Several studies suggest that viral respiratory infections significantly alter the lung environment, creating conditions favorable for bacterial colonization and subsequent patient disease exacerbation [155]. For example, RSV infection has been shown to increase the binding of *P. aeruginosa* to primary lung epithelial cells in vitro, including through the promotion of biofilm formation [156]. The impaired immune response following viral infections, also observed in other types of chronic respiratory diseases (e.g., asthma and COPD), may also facilitate secondary bacterial colonization contributing to disease severity [155]. Surprisingly, the scenario of chronic primary bacterial colonization, which emphasizes the impact of secondary viral infection, a case frequently encountered in CF, remains relatively unexplored in the literature. The associated molecular mechanisms at the level of cellular signaling pathways are not yet fully elucidated. A comprehensive review of virus/bacteria co-infections in the context of CF has recently been published by Kiedrowski and Bomberger [157].

Both viral and bacterial infections are also associated with exacerbations of chronic obstructive pulmonary disease (COPD) [158]. A wide variety of respiratory viruses have been implicated as playing a role in bacterial exacerbations of COPD due to the *H. influenzae*, *M. catarrhalis*, *S. aureus*, *P. aeruginosa*, and *Enterobacter spp.* In line with these observations, a recent study found that 15 days after experimental rhinovirus infection of subjects with COPD, there was a sixfold increase of the 16S copy number detected in sputum compared to baseline values obtained from sputum collected before RV inoculation [159]. The succession of viral and/or bacterial infections during childhood, and their long-term consequences on the immune response, could constitute a favorable ground for the development of asthma. The involvement of bacteria such as *S. pneumoniae* or *H. influenzae* and recurrent viral infections by RSV have been described and considered critical factors [160,161]. Nevertheless, there is currently little information on a specific contribution to viral/bacterial co-infections.

## 4. What about the Microbiome?

For a long time, the respiratory tract was considered a sterile environment, and the presence of bacteria was associated with acute or chronic infection. The discovery of a respiratory microbiome started with the Human Microbiome Project in 2008, thanks to the development of high-throughput sequencing using the 16S rRNA gene [162]. Since then, various studies have demonstrated the presence of microbiome in the upper respiratory tract (URT) and lower respiratory tract (LRT) and its importance for lung homeostasis [163]. Since the last decade, numerous studies were performed to understand the influence of viral infection on the microbiome. Various NGS studies demonstrated that RSV and influenza virus altered the respiratory microbiome through direct or indirect processes, inducing a dysbiosis [164,165,166,167]. Metagenomic studies performed on infected human samples by respiratory virus demonstrated a shift from Bacteroidetes to Proteobacteria, including numerous Gram-negative pathogenic bacteria [167,168].

So far, bacterial colonization of the URT is considered as the first stage of bacterial invasion in the lungs after primary viral infection. It has been demonstrated that influenza virus and RSV enhance bacterial adherence and colonization, leading to secondary infection and pneumonia [58,70]. Besides, influenza virus also promoted S. pneumoniae transmission in mice [169]. Moreover, influenza-virus-induced signals, such as the release of ATP, lead to increased dispersed bacteria from biofilm, associated with a pathogenic phenotype [170,171]. Using RNA-seq, Pettigrew and collaborators characterized the effect of influenza virus on *S. pneumoniae*. An enhanced expression of several genes involved in bacterial metabolism and motility was observed in dispersed bacteria, suggesting that influenza virus could directly or indirectly influence commensal *S. pneumoniae* phenotype [172]. Another possibility could be that by altering the microbiome, a viral infection might decrease bacteria species that usually keep at bay pathogenic bacteria and thus, protect the host. Discrepancies exist about the respiratory microbiome and the disease severity. Some studies demonstrated an association between increased diversity and influenza or RSV infection severity, while others showed the contrary. Besides, divergent results were observed about the association of dysregulation of microbiome species and viruses. For example, some studies revealed a positive association between RSV and *H. influenzae* or the influenza virus and *S. aureus*, while others failed to observe this effect [166].

Viral infections might also induce a different host state that promotes microbiome alteration and secondary bacterial infection. During viral infection, the increase of inflammation through the production of cytokines could create a suitable environment for proliferation of some commensal bacteria, such as *S. pneumoniae* or *S. aureus*. Type III IFN induced after influenza infection was associated with a restructuring and expansion of the nasal microbiome, as observed for *Klebsiella*. Moreover, an increase of secondary infection by *S. aureus* was also observed after viral infection, suggesting an interplay between microbiome and pathogens during co-infections [137,138]. Until then, studies of co-infections were mainly based on the interaction between two pathogens without considering the lung microbiota. However, it is crucial to consider the respiratory microbiome as it could influence the mechanism leading to co-infections and could be a therapeutic target. Recently, Kanmani and collaborators observed the interplay between co-infection and the microbiome. Using a commensal nasal bacterium (*C. pseudodophteriticum*) as a probiotic in the infant mouse model, they observed an improved antiviral immune response against RSV and a subsequent infection by *S. pneumoniae* [173]. Other studies demonstrated bacteria interspecies competition, which could alter co-infection between virus and bacteria [166].

## 5. What Are the Best Models for Studying Co-Infections?

Many different models have been used to study co-infections (Table 1); each model has advantages and disadvantages, depending on the nature of the pathogens studied and the experimental objectives. It is therefore difficult to say which model is the best, as they are all very complementary.

### 5.1. In Vitro Models

Compared to the large number of clinical studies or in vivo experiments, there are relatively few experimental models of in vitro co-infections/superinfections described in the literature. Several in vitro superinfection models using monolayer cell lines or primary cells derived from epithelia or macrophages have been proposed—influenza/*S. aureus* in A549 cells [174,175], or RSV/*S. pneumoniae* in primary macrophages [128] provide important information on the host response and mutual virus/bacteria interactions. Although essential, these models present some limitations. Beyond the biological relevance, which may be questionable for some cell lines, the mode of infection/superinfection of bacteria in the cell supernatant (planktonic bacteria) may not always represent what happens in patients. To study co-infections/superinfections in a much more physiological context, several teams are now working with reconstituted human epithelial models, consisting of primary differentiated respiratory cells grown at the air-liquid interface [129]. These experimental models allow a much more integrated approach, as it is possible to simultaneously study the impact of co-infections/superinfections on the physiology of the epithelium (monitoring of trans-epithelial resistance), but also on the immune response (measurement of cytokines/chemokines at the apical/basal level) or, more broadly, on the global response of the host (transcriptome/proteome, etc.). Other, more complex models, such as lung-on-chip or lung-organoids (reviewed in [185]), are also promising models for studying co-infections. These models are still quite costly and require significant expertise, limiting their use on a large scale.

### 5.2. In Vivo Models

Laboratory animal models have a crucial role in pre-clinical studies for vaccines or therapeutics approaches, especially for assessing efficacy and safety, but also essential to studying pathogenesis and transmission of pathogens. A good model needs to simulate, pathogen replication, host response, and clinical signs as closely as possible to what would happen in humans. However, there are various points to keep in mind in the choice of the model, such as study goals, availability of reagents, and animal background [186].

#### 5.2.1. Mouse Model

The mouse model is widely used to model infectious diseases. Its main advantage is practicality with low cost, size, husbandry requirements, the availability of reagents, and the possibility of using transgenic, humanized, or knock-out mice to study different host responses. However, this model also presents disadvantages such as semi-permissive replication or the need for strain adaptation to induce viral replication and disease in the lungs, or the absence of viral receptors, as well as different signs of disease compared to human disease and the influence of genetic background [187,188,189,190]. Different superinfection models were developed, especially with influenza virus and secondary infection with *S. pneumoniae* or *S. aureus*. Mice primarily infected with influenza and subsequently with *S. pneumoniae* or *S. aureus* mimicked very well what is observed in children with a mixed respiratory infection. Synergism was observed between both pathogens, with an increase in morbidity and mortality associated with higher bacterial load in the lungs and increased lung injury. On the contrary, simultaneous infection presented an additive effect, suggesting that interval for sequential infection is decisive. Finally, primary bacterial infection prevented secondary infection by influenza, suggesting the importance of the order of the pathogens, with the underlying mechanisms explaining these differences remaining to be better characterized. Mouse models were also developed to study co-infection between RSV and *S. pneumoniae* or *S. aureus*. Similarly, influenza mixed infection resulted in higher susceptibility to secondary infection in mice. However, simultaneous infection of RSV and *S. pneumoniae* or *S. aureus* in mice resulted in higher bacterial load than sequentially infected mice, suggesting different outcomes depending on the pathogens [46,59,113,176,177]. Altogether, these studies demonstrated that the mouse model is an attractive model to study mixed infections. So far, all the data available about the effect of dual infection on the host immune response were obtained using the mouse model, thanks to transgenic and knock-out mice [45,64,72,178,179,180]. 

#### 5.2.2. Non-Human Primate Model

Due to physiology and genetic similarities, non-human primates (NHP) are the closest model to study human respiratory viruses. They present similar morphology and reproduce the features of human pneumonia. However, high cost, practical aspects, and ethical considerations lead NHP to be less accessible. Different NHP species models have been developed for RSV, influenza, and SARS-CoV-2, and simple bacterial infection with different results depending on the species [188,189,190,191,192]. The first study of dual respiratory infection in NHP was performed in squirrel monkeys infected with influenza virus following by *S. pneumoniae* four days later. Primary viral infection potentiated secondary bacterial infection, leading to severe pneumonia and high bacterial load in the lungs compared to single-infected animals [44]. More recent studies demonstrated similar results in infection various influenza strains, such as H3N2, H1N1, and H7N7 associated with *S. aureus*. Mixed infection increased morbidity and mortality compared to single infection, and results in NHP were more consistent with dual infection observed in healthy humans [43,181]. Vaccination against H7N7 prevented disease by decreasing morbidity associated with reduced viral replication and, to a lesser extent, bacterial load, suggesting a role of vaccine to minimize co-infections [182]. So far, no model exists for mixed infection involving RSV or SARS-CoV-2 with bacteria.

#### 5.2.3. Ferret Model

Ferrets are a valuable model for studying human respiratory viruses, as similar pathology has been observed in different infection models. For influenza viruses, the main advantage is that adaptation is not required to induce disease in the animals. However, a major drawback of this model is the limited availability of reagents and immunological tools to study the response to infection. Recently, this model was also used to study human RSV, but only mild clinical signs were recorded. Finally, experimental infection by SARS-CoV-2 demonstrated that ferrets might not be the most relevant with a predominant upper-respiratory tract infection [190,193]. Ferrets have been used to study bacterial-related pneumonia, such as *S. pneumoniae* or *S. aureus,* as well as *P. aeruginosa* in the case of cystic fibrosis [194,195,196]. Enhanced bacterial adherence was observed in the nasal mucosa of influenza-infected ferrets, suggesting a synergism between those two pathogens in this model [183]. As observed in humans, more severe illness was observed in mixed infection compared to the simple one, with a higher bacterial load in the upper respiratory tract. Similarly, in human disease, mild to severe histopathology lesions were observed. Depending on influenza viral strain or subtype, the outcome of secondary infection was different, as H3N2 induced more severe sinusitis compared to influenza B virus (IBV), for example, thus supporting epidemiological data [194]. Moreover, the pneumonia severity of dual infection was also dependent on *S. pneumoniae* strains [197]. 

#### 5.2.4. Other Models of Interest

Other models have also been used the study the importance of co-infections in respiratory disease. Nguyen and collaborators developed a co-infection model in cotton rats to understand the interaction between RSV and *S. pneumoniae*. Primary bacterial colonization followed by RSV infection led to an increased viral replication in the respiratory tract, but with dependence on *S. pneumoniae* strains [198]. Higher bacterial load and lung injury were also observed in cotton rats infected with influenza and *S. aureus*; however, no clinical signs were observed in single or co-infected animals [199]. Other studies used the chinchilla model to study otitis media in mixed infections involving RSV or influenza viruses. Primary viral infection enhanced the severity of the disease by promoting bacterial replication [200,201]. Human challenges have been performed since the 1930s, mainly to characterize influenza infection, but they became rare and challenging to set up in the 1990s due to more strict regulations [184]. Recently, Jochems and collaborated used a human model challenge to assess host change in mixed influenza virus and *S. pneumoniae* infection in the nasal tract. Fifty-three volunteers were infected with a live-attenuated influenza virus (LAIV) and three days later with *S. pneumoniae*. Viral infection increased bacterial acquisition and carriage compared to simple infection. Moreover, higher production of cytokines such as IP-10, IL-15, IL-10, or IFN-γ was associated with mixed infection. Analysis of host gene signatures revealed an alteration of innate immune genes such as PRRs, type I and II IFN, or interleukins, underlining that LAIV improved secondary bacterial infection by altering the host response. Surprisingly, contrary to the mouse model, type 17 response was not involved in higher susceptibility to mixed infection, demonstrating that animal models might not reflect human disease [202]. 

## 6. Concluding Remarks

Respiratory tract co-infections are a model of complex functional interactions between viruses/bacteria and host cells. The nature of the different pathogens involved, the microbiological and cellular contexts, the sequence and timing of infections are essential parameters that determine the underlying physiological and immunological mechanisms of disease severity. Given this complexity, at the fundamental level, to deepen our understanding of the role of co-infections in the aggravation of respiratory pathology, it will be necessary to combine different techniques in an integrated approach, trying to work on the most relevant experimental models in terms of physiology. Some specific areas of research probably deserve particular attention. Future work is needed to better understand, for example, the key role played by the sequence and timing of infections, or the detrimental role of the innate immune response often observed in bacterial superinfections. In addition to deciphering the mechanisms underlying the severity, the study of co-infections of the respiratory tract is also likely to provide new diagnostic and therapeutic solutions. The identification of biomarkers of severity, for example, could allow more rapid and effective management of patients. At the same time, host-targeted therapeutic approaches could qualify for more appropriate treatment of co-infections, less likely to be subject to the emergence of antiviral and/or antibiotic resistance. Respiratory co-infections remain an unexplored territory, and their study will undoubtedly bring us many surprises and hopefully new tools to fight them.

## Figures and Tables

**Table 1:** Overview of the available experimental models for the study of viral/bacterial co-infections, with their respective advantages and drawbacks.

Experimental Models		Advantages	Drawbacks	Refs
In vitro	Cell monolayers (Cell lines/primary cells)	Easy handling/practical Low cost	Limited biological relevance	[128,174,175]
	Reconstituted HAE	Physiological relevance	High cost	[129]
In vivo	Mouse	Low cost Availability Transgenic/Humanized/KO models	Limited biological relevance	[45,46,59,113,176,177,178,179,180]
	NHP	Physiological relevance	High cost Ethical concern High complexity Availability of reagents	[43,44,181,182]
	Ferret			[183]
	Human challenge			[184]
